# Dual Control of Giant Field-like Spin Torque in Spin Filter Tunnel Junctions

**DOI:** 10.1038/srep11341

**Published:** 2015-06-22

**Authors:** Y. -H. Tang, F. -C. Chu, Nicholas Kioussis

**Affiliations:** 1 Department of Physics, National Central University, Jung-Li 32001, Taiwan; 2 Department of Physics, California State University, Northridge, CA 91330-8268, USA

## Abstract

We predict a giant *field-like* spin torque, 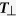, in spin-filter (SF) barrier tunnel junctions in sharp contrast to existing junctions based on nonmagnetic passive barriers. We demonstrate that 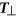 has linear bias behavior, is independent of the SF thickness, and has odd parity with respect to the SF’s exchange splitting. Thus, it can be selectively controlled via external bias or external magnetic field which gives rise to sign reversal of 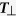 via magnetic field switching. The underlying mechanism is the interlayer exchange coupling between the *noncollinear* magnetizations of the SF and free ferromagnetic electrode via the nonmagnetic insulating (I) spacer giving rise to *giant* spin-dependent reflection at the SF/I interface. These findings suggest that the proposed field-like-spin-torque MRAM may provide promising dual functionalities for both ‘reading’ and ‘writing’ processes which require lower critical current densities and faster writing and reading speeds.

Recent discoveries in the ferromagnet/insulator/ferromagnet (FM/I/FM) magnetic tunnel junctions (MTJs) have demonstrated that the relative orientation of the two FM electrodes can be either altered by an external magnetic field, i.e. the tunneling magnetoresistance (TMR) effect^1^, or controlled by a spin-polarized current, i.e. the current-induced magnetization reversal via the spin transfer torque (STT) effect^2^,^3^. The spin-transfer, 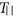, and field-like, 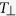, components of the STT originate from different components of the spin current accumulated at the FM/I interface^4^,^5^ and can be expressed in terms of the interplay of spin current densities^6^,^7^ and of the non-equilibrium interlayer exchange couplings^8^, respectively, solely in collinear configurations.

Usually, the writing process in magnetic random access memory (MRAM) bits is performed via the spin transfer torque, 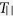
9,10,11, which is much larger than the field-like component, 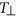, while the read-out operation is reliably performed via the TMR effect^12^. However, the magnetization switching requires high current densities and hence high power consumption, both of which are detrimental also to the TMR. Therefore, alternative writing and reading mechanisms for MTJs may provide a viable route towards switching energies per bit close to or smaller compared with CMOS (~1 fJ)^13^.

The insulator in conventional FM/I/FM MTJs plays only a passive role in the spin-polarized transport. The evolution beyond passive components has broadened the quest for multifunctional spintronic devices consisting of either ferroelectric^14^,^15^,^16^,^17^,^18^,^19^ or spin-filter (SF) barriers^20^,^21^,^22^,^23^. The latter exploits the separation of the barrier heights, *φ*_*σ*_, of the two spin channels, 2Δ ≡ *φ*_↑_ − *φ*_↓_, which can be in turn tuned via an external magnetic field^20^,^23^. Early SF tunnel junction structures employed the europium chalcogenides (EuS, EuO, etc.) as a ferromagnetic barrier^20^,^21^,^22^,^24^. The limitation of low Curie temperatures for this family of compounds sparked intense research interest in perovskite- and spinel-structured ferrite and manganite compounds, such as NiFe_2_O_4_^25^, CoFe_2_O_4_^26^, NiMn_2_O_4_^27^, and BiMnO_3_^28^, which magnetically order at much higher temperatures. The large TMR values reported^29^ in NM/SF/FM MTJs, where NM denotes a nonmagnetic metal, result from the combination of the spin filter tunnel barrier and the ferromagnetic electrode. Furthermore, NM/SF/I/FM MTJs consisting of a thin nonmagnetic insulator spacer to effectively decouple the SF-barrier and the FM electrode exhibit large magnetoresistance^30^. In double SF tunnel junctions NM/SF/I/SF/NM proposed by Miao *et al.*^31^,^32^, the tunneling probabilities for spin-up and spin-down electrons are different because they depend exponentially on the spin-dependent barrier height. By toggling the magnetization of the two SF barriers between parallel and antiparallel configurations a high TMR value was achieved.

The objective of this work is to employ tight binding calculations and the non-equilibrium Green’s function formalism to study the effect of the SF-barrier magnetization on the bias behavior of both components of STT in noncollinear FM/I/SF/I/FM junctions. We predict a *giant* field-like spin torque component, 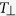, in contrast to conventional FM/I/FM junctions, which has linear bias dependence, is independent of the SF thickness, and has sign reversal via magnetic field switching. The underlying mechanism is the interlayer exchange coupling between the *noncollinear* magnetizations of the SF and free ferromagnetic electrode via the nonmagnetic insulating spacer giving rise to *giant* spin-dependent reflection at SF/I interface. We demonstrate dual manipulation of 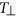 via external magnetic field and external bias which provides a new avenue to achieve both ‘reading’ and ‘writing’ processes of nonvolatile field-like spin torque MRAM (FLST-MRAM), which may require lower critical current densities for magnetization switching than conventional STT-MRAM.

## Results

**Figure 1:**
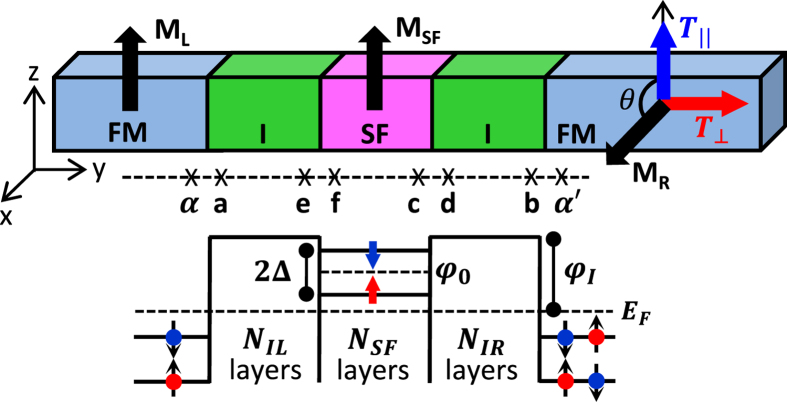
Schematic of FM/I/SF/I/FM junction consisting of left and right semi-infinite FMs sandwiching the central region of the left and right nonmagnetic insulators and the SF. *N*_*IL*(*IR*)_ and *N*_*SF*_ denote the atomic layers in the left (right) I and central SF, respectively. The spin-polarized barrier heights of the SF are 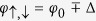 and of the I is *φ*_*I*_, respectively. The magnetization of the SF, **M**_**SF**_, can be toggled between the ±z direction under an external magnetic field. The magnetization of the left (fixed) FM, **M**_**L**_, is pinned along the z direction, while that of the right (free) FM, **M**_**R**_, is rotated by the angle *θ* = π/2 around the y axis with respect to **M**_**L**_.

We consider the Co/Al_2_O_3_/EuS/Al_2_O_3_/Co junction, shown schematically in Fig. 1, consisting of three layer I/SF/I barrier sandwiched between two semi-infinite FM electrodes. The two nonmagnetic insulators serve as spacer layers between the Co and the SF to prevent any direct exchange magnetic coupling and to ensure independent switching of the SF magnetization, **M**_**SF**_, or the magnetization of the right FM electrode, **M**_**R**_. The ferromagnetic ordering in europium chalcogenides originates from the localized moments of the Eu 4*f*-derived states which in turn causes a large exchange splitting, 2Δ, between the Eu-5*d* majority- and minority-derived conduction bands^33^. The direction of the in-plane magnetization **M**_**SF**_ can be toggled along the ±*z* direction by an external magnetic field^20^,^23^ which induces in turn sign reversal of Δ.

The spin-transfer, 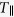, and field-like, 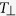, components of the *net* spin torque per interfacial unit area, ?, on the right (free) FM are along the 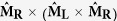 and the 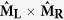 directions, respectively, shown in Fig. 1. Here, 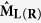 is the unit vector of the magnetization of the left (right) FM. These can be determined from the spin current density accumulation at the right I/FM interface^7^,
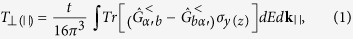
where 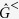 is the 2 × 2 Keldysh Green’s function matrix in spin space, *α*′ and *b* are the first and last sites of the right FM electrode and the right I-barrier, respectively, shown in Fig. 1, *σ*_*y*(*z*)_ is the y (z)-component of the Pauli matrix vector, 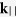 is the transverse wave vector, and the energy integral is over occupied states.

Extending the non-equilibrium Keldysh formalism for the conventional FM/I/FM in the limit of thick barrier^34^,^35^ to the FM/I/SF/I/FM junction we find that the Green’s function matrix at the right I/FM interface can be written as
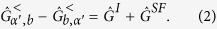


Here, 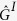, is the Keldysh Green’s function for the entire junction which is analogous to Eq. (9) in Ref. 34 for the FM/I/FM junction. However, the additional term, 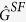, arises from the spin-dependent reflection at the SF/I interface due to tunneling electrons from the right FM. The 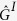 and 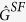 are given by
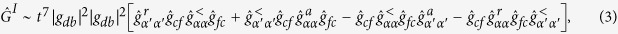
and
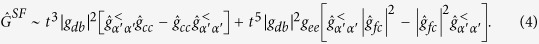


Here, the subscripts refer to the sites in the various regions of the FM/I/SF/I/FM junction in Fig. 1, 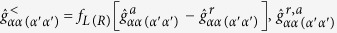 are the retarded and advanced surface Green’s function matrices of the isolated left (right) FM, and *f*_*L*(*R*)_ is the Fermi-Dirac distribution function of left (right) FM electrode. The Green’s functions, 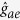, 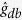, and 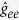, of the isolated I-barrier are real, and the Green’s function matrices, 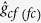 and 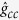, of the isolated SF-barrier are real and diagonal.

Assuming that the magnetizations of the left fixed ferromagnet, **M**_**L**_, and the spin filter, **M**_**SF**_, are collinear and substituting Eqs. (3) and (4) in Eq. (1) we find that the STT componen.ts are


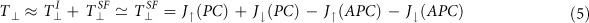


and


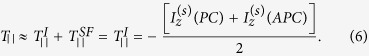


Note that the effect of SF dominates 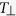 while is negligible for 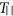, because the Im[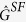] has only nonzero off-diagonal matrix elements. Here, *J*_↑(↓)_ is the non-equilibrium interlayer exchange coupling (NEIEC) between the spin-↑(↓) states of the left and right FMs in the parallel (PC) and anti-parallel (APC) configurations, and 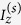 is the spin current density along *z* for the PC and APC. These general expressions demonstrate that the bias dependence of noncollinear 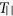 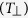 components of the STT can be decomposed as the interplay between 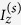 (NEIECs) solely in the collinear magnetic configurations. This in turn allows the efficient calculation of the STT from *collinear ab initio* electronic structure calculations^36^,^37^. We would like to emphasize that the results of the calculations are general and do not depend whether the *collinear* magnetizations of the left fixed ferromagnet and the spin filter are out-of-plane or in-plane.

**Figure 2:**
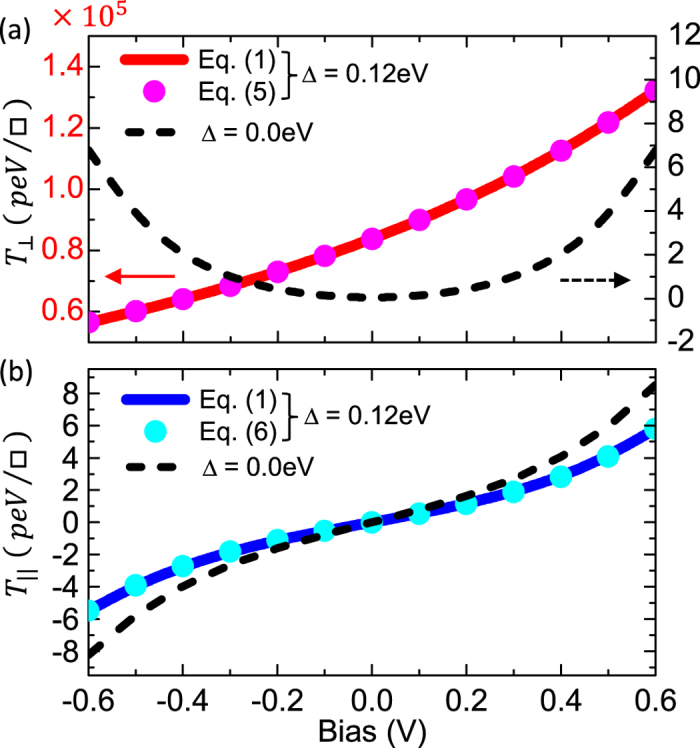
Bias dependence of (**a**) 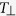 and (**b**) 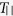
 for the FM/I/SF/I/FM junction with Δ = 0.12 eV and N_*IL*_ = N_*SF*_ = N_*IR*_ = 3. The solid curves and circles represent the STT component calculated by Eq. (1) and Eqs. (5) and (6), respectively. The dashed curves denote the STT components for the conventional FM/I/FM junction with *N*_*I*_ = 9 and Δ = 0.0 eV. Note the different scale in panels (**a**) and (**b**).

In Fig. 2 we present the bias dependence of 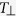 and 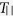 for the FM/I/SF/I/FM junction with Δ = 0.12 eV and N_*IL*_ = N_*SF*_ = N_*IR*_ = 3. The solid curves and circles represent the STT values calculated from Eq. (1) and Eqs. (5) or (6), respectively, demonstrating the excellent agreement between these two computational schemes. We also show for comparison the STT components (dashed curves) for the conventional FM/I/FM junction with *N*_*I*_ = 9, i.e., the same thickness of the I-barrier, and Δ = 0.0 eV. Note the different scales in the left- and right-hand ordinates in Fig. 2(a). The most striking result is the *giant* values of 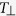 in the SF-junction which is about *four orders* of magnitude higher than 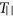, in sharp contrast to conventional FM/I/FM junctions where 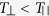. Furthermore, the SF renders the bias behavior of 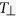 nearly linear in the low bias regime while in conventional FM/I/FM junctions is purely quadratic. On the other hand, the effect of the SF on the bias behavior of 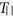 is small compared to that in the conventional junction with Δ = 0.0 eV, due to the fact of 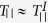. This giant enhancement of the field-like torque may in turn lead to reduction of the critical current necessary for magnetization switching in the next-generation MRAMs. However, the resistance-area product (RA) (and hence the barrier thickness) in MTJs used for MRAM will also have an important role on the write energy per bit and the switching current density^38^.

**Figure 3:**
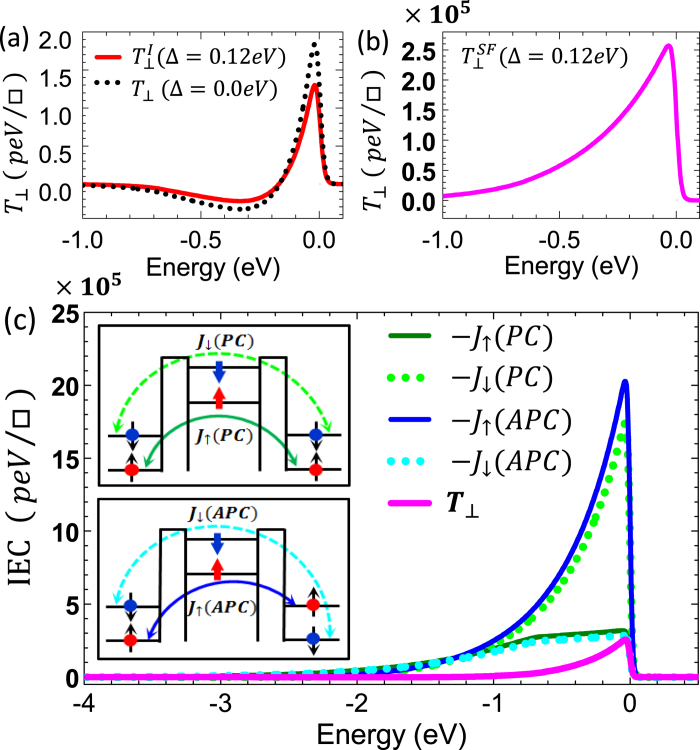
(**a**) Zero-bias energy-resolved field-like component of the spin torque (dotted black curve) 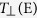 ≡ 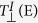 for a conventional FM/I/FM junction with Δ = 0.0 eV and *N*_*I*_ = 9 compared to the energy-resolved contribution, 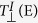, [Eq. (5)] from the non-magnetic insulator spacer (red solid curve) for the FM/I/SF/I/FM junction. (**b**) Zero bias energy-resolved contribution, 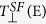, from the spin filter barrier [Eq. (5)] (magenta curve) for the FM/I/SF/I/FM junction. Note the different scale in panels (**a**) and (**b**). (**c**) Zero bias energy-resolved interlayer exchange couplings between the left fixed and right free ferromagnets in the PC and APC configurations [Eq. (5)], shown schematically in the inset, for the FM/I/SF/I/FM junction. In all panels the Fermi energy is at 0.0 eV and for the FM/I/SF/I/FM junction Δ = 0.12 eV and N_*IL*_ = N_*SF*_ = N_*IR*_ = 3.

In order to elucidate the underlying mechanism of the SF-induced enhancement of 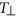, we show in Fig. 3(a,b) the zero-bias energy-resolved contributions of the nonmagnetic insulator spacer, 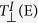, and of spin-filter, 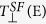, respectively, to the *net* field-like spin torque, 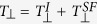, in Eq. (5). 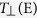 is the energy-dependent integrand in Eq. (1) after integrating over the transverse wave vector, 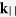. For conventional FM/I/FM junction (Δ = 0) the spin-filter contribution, 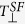, vanishes identically and 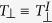. On the other hand, for the FM/I/SF/I/FM junction (Δ ≠ 0) the important question is what is the relative size of 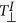 and 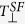
? Interestingly, for the FM/I/SF/I/FM junction we find that the energy-resolved contribution, 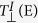, from the nonmagnetic insulator spacer [red solid curve in Fig. 3(a)] is qualitatively similar to, 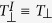, for a conventional FM/I/FM junction (Δ = 0) [dotted black curve in Fig. 3(a)]. In sharp contrast, the SF energy-resolved contribution, 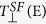, [magenta curve in Fig. 3(b)], which is present solely in SF-based MTJ is *five orders of magnitude* larger than that of the non-magnetic insulator spacer, 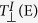, [note the difference in scale in Fig. 3(a,b)]. The giant value of 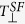 [magenta curve in Fig. 3(b)] arises from the additional term, 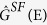, in Eq. (4) due to the spin dependent reflection at the SF/I interface which is associated solely with the interlayer exchange coupling (IEC) between the SF and the right (free) FM electrode. Namely, because 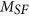 is *noncollinear* with 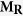 only the spin-polarized electrons from the right FM encounter the *non-collinear* exchange field of the SF-barrier, thus giving rise to giant spin accumulation at the right I/FM interface.

Fig. 3(c) displays the energy dependence of the four IECs in the PC and APC configurations, shown schematically in the inset, as well as 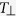 at zero bias for the SF-based junction with Δ = 0.12 eV and N_*IL*_ = N_*SF*_ = N_*IR*_ = 3. For both PC and APC we find that 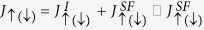, which is induced primarily from the giant IEC between the *noncollinear* SF barrier and the right FM due to the spin-dependent reflection at the SF/I interface. It is interesting to note that 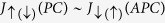 because they represent the IEC of the majority band in the right FM with the majority and minority conduction bands of the SF barrier, respectively, shown in the inset of Fig. 3(c).

## Discussion

**Figure 4:**
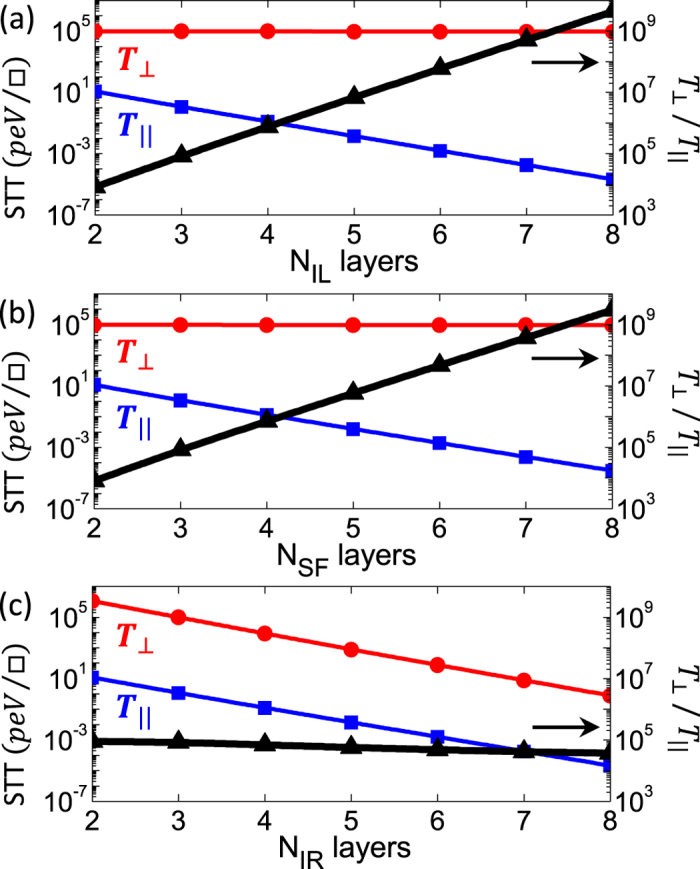
Spin-transfer, 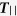, (blue curves), field-like, 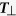 , (red curves), components of spin transfer torque per unit interfacial area, and their ratio, 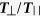, (black curves) plotted in logarithmic scale (left-hand ordinate for 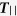 and 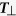 and right-hand ordinate for 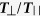) as a function of the number of atomic layers in the (**a**) left I-barrier (N_*IL*_), (**b**) SF-barrier (N_*SF*_), and (**c**) right I-barrier (N_*IR*_) for the FM/I/SF/I/FM junction with Δ = 0.12 eV under an external bias of 0.2 V.

We first examine the effect of barrier thickness on both spin torque components for SF-based junctions. In Fig. 4 we show 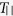 (blue curves), 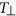 (red curves), and the ratio 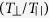 (black curves) on a logarithmic scale as a function of the number of atomic layers in the (a) left I-barrier (N_*IL*_), (b) SF-barrier (N_*SF*_), and (c) right I-barrier (N_*IR*_) under 0.2 V external bias. Interestingly, similar to conventional FM/I/FM junctions, 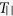 decays exponentially as the number of layers in the insulating and SF barriers increases with the same decay rate. In sharp contrast, the field-like component, 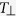, exhibits a weak thickness dependence on the left insulating spacer and the SF barrier while it shows a strong exponential decay on the thickness of the right insulating spacer similar to that of 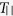. This is due to the fact that 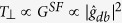 of the right I-spacer which depends exponentially on *N*_*IR*_ and is nearly independent of the thickness of the left I-barrier and the SF. Consequently, the ratio 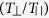 increases exponentially with N_*IL*_ and N_*SF*_, while it remains approximately constant (~10^4^) as N_*IR*_ increases. These intriguing findings pave the way towards novel opportunities for the next-generation of multifunctional non-volatile memories based on *field-like spin torque* MRAM (FLST-MRAM), where the ‘writing’ processes can be achieved by manipulating 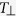 via lower current densities. This in turn may resolve the bottleneck of high writing current densities required in the existing STT-MRAMs.

**Figure 5:**
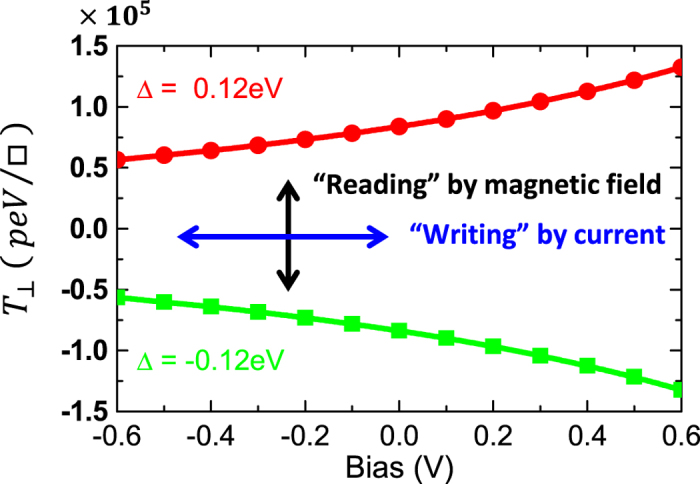


Figure 5 shows the bias dependence of 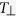 in the SF-based tunnel junction with Δ = ±0.12 eV. Interestingly we predict that the field-like spin torque can be switched via a sign reversal of the SF’s exchange splitting, Δ, which can be achieved under reversal of the direction of an external magnetic field^20^,^23^, i.e. 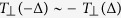. This stems from the fact that the four individual NEIECs in Eq. (5) satisfy the relations: 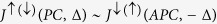 and 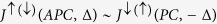, as can be inferred from the inset in Fig. 3(c). These results demonstrate the *dual* control of the giant field-like STT either via current or magnetic field which reverses the exchange splitting of the SF-barrier. The lower coercive field of EuS thin films (~60–150 Oe^24^) compared to that of the free FM film (~400–600 Oe for FeCo) may allow the magnetization switching of the SF magnetization via an external magnetic field without affecting the magnetization of the right FM film. Thus, the dual control of 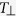 provides promising novel functionalities for both ‘reading’ and ‘writing’ processes in the newly proposed non-volatile FLST-MRAMs.

**Figure 6:**
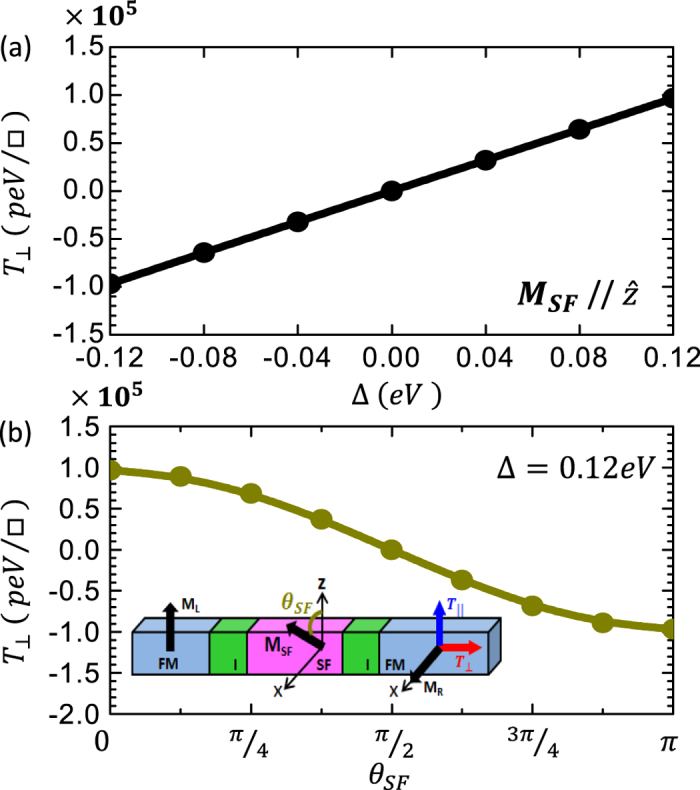
(**a**) 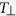 for the FM/I/SF/I/FM junction as a function of the exchange splitting, Δ, of the SF barrier, where **M**_**SF**_ is along the z-direction as shown schematically in Fig. 1 (b) Angular dependence of 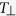 for the FM/I/SF/I/FM junction, where *θ*_*SF*_ is the angle between **M**_**SF**_ and the easy *z*-axis as shown schematically in the inset. In both panels N_*IL*_ = N_*SF*_ = N_*IR*_ = 3 and the external bias is 0.2 V.

Figure 6(a) shows the variation of the field-like spin torque for the FM/I/SF/I/FM tunnel junction versus the exchange splitting, Δ, of the SF barrier which can be tuned, for example, by an external magnetic field. We find that 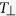 varies linearly with the exchange splitting and its giant value remains robust provided that Δ ≠ 0. Note, that for Δ = 0 the field-like torque is reduced by four orders of magnitude. In order to examine the robustness of the giant field-like spin transfer torque against small fluctuations of the in-plane SF magnetization, **M**_**SF**_, from the easy *z*-axis, we show in Fig. 6(b) 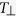 as a function of the angle, *θ*_*SF*_, of the SF magnetization with respect to the *z*-axis in the x-z interfacial plane [inset in Fig. 6(b)]. We find that even though 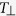 decreases with increasing *θ*_*SF*_, its giant value remains robust except for 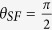, where the magnetization of the right FM becomes collinear with the magnetization of the SF, and the giant spin accumulation at the right I/FM interface is reduced dramatically by four orders of magnitude.

In summary, we predict a *giant* field-like spin torque in FM/I/SF/I/FM junctions which has linear bias behavior, is independent of the SF-barrier thickness and whose sign can be toggled by switching the SF magnetization direction under an external magnetic field. These findings are in sharp contrast to those in conventional tunnel junctions based on nonmagnetic passive barriers where 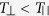, 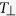 has a quadratic bias behavior and decreases exponentially with the barrier thickness. The underlying origin is the *giant* interlayer exchange coupling between the *noncollinear* magnetization of the SF and free ferromagnetic electrode via the nonmagnetic insulator spacers. Our results suggest that the novel dual manipulation of 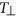 either by a magnetic field or bias can be employed for ‘reading’ or ‘writing’ processes, respectively, in the next-generation FLST-MRAMs. We hope these predictions inspire further experimental explorations of STT in SF-based junctions, especially in Fe/MgO/EuO/MgO/Fe MTJs where one can exploit both the central SF barrier and the spin filter effect of the Fe/MgO stack on the Fe majority spin electrons at the Fermi energy with Δ_1_ symmetry.

## Method

The Hamiltonian for the FM/I/SF/I/FM junction is described by the tight binding Hamiltonian^7^,^35^, *H* = *H*_*L*_ + *H*_*R*_ + *H*_*C*_ + *H*_*cpl*_, where *H*_*L*_, *H*_*R*_, *H*_*C*_ are the Hamiltonian of the isolated left, right, and central (I/SF/I) region, respectively, and *H*_*cpl*_ is the coupling between the electrodes and the central scattering region. Within the single-band tight-binding model^34^ the Co majority- and minority-spin-dependent on-site energies are *ε*^↑^ = 1.0 eV and *ε*^↓^ = 2.5 eV, respectively, which describe correctly the position and exchange splitting of the Δ_1_ band in Co. The spin-dependent onsite energy of the EuS barrier are 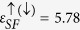
eV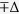 with Δ = 0.12 eV. The spin-independent onsite energy for the Al_2_O_3_ is *ε*_*I*_ = 5.98 eV and the nearest-neighbor hopping matrix element is *t* = −0.83 eV in all regions. These parameters are chosen to describe correctly (i) the average SF barrier height *φ*_0_ = 0.8 eV, (ii) the insulating barrier height *φ*_*I*_ = 1.0 eV, and (iii) the exchange field Δ = ±0.12 eV in Al/EuS/Al_2_O_3_/Co^30^. However, the results do not depend on intricate details of the Co band structure and can be considered to be generally valid for partially spin-polarized FM, such as Fe or SrRuO_3_. The effect of external bias, V, is to shift the chemical potential of the right electrode with respect to that of the left electrode, *μ*_*R*_ − *μ*_*L*_ = *eV*, and *μ*_*L*_ is fixed at the Fermi energy, *E*_*F*_ = 0.0 eV.

## Additional Information

**How to cite this article**: Tang, Y. -H. *et al.* Dual Control of Giant Field-like Spin Torque in Spin Filter Tunnel Junctions. *Sci. Rep.* **5**, 11341; doi: 10.1038/srep11341 (2015).
